# Large Italian Multicenter Study on Prognostic Value of Baselines Variables in mCRPC Patients Treated with ^223^RaCl_2_: Ten Years of Clinical Experience

**DOI:** 10.3390/diagnostics15030339

**Published:** 2025-01-31

**Authors:** Maria Silvia De Feo, Luca Filippi, Matteo Bauckneht, Elisa Lodi Rizzini, Cristina Ferrari, Valentina Lavelli, Andrea Marongiu, Gianmario Sambuceti, Claudia Battisti, Antonio Mura, Giuseppe Fornarini, Sara Elena Rebuzzi, Alessio Farcomeni, Alessandra Murabito, Susanna Nuvoli, Miriam Conte, Melissa Montebello, Renato Patrizio Costa, Arber Golemi, Manlio Mascia, Laura Travascio, Fabio Monari, Giuseppe Rubini, Angela Spanu, Giuseppe De Vincentis, Viviana Frantellizzi

**Affiliations:** 1 Department of Radiological Sciences, Oncology and Anatomo Pathology, Sapienza, University of Rome, 00161 Rome, Italy; mariasilvia.defeo@uniroma1.it (M.S.D.F.); miriam.conte@uniroma1.it (M.C.); melissa.montebello@uniroma1.it (M.M.); giuseppe.devincentis@uniroma1.it (G.D.V.); viviana.frantellizzi@uniroma1.it (V.F.); 2 Department of Biomedicine and Prevention, University of Rome “Tor Vergata”, Via Montpellier 1, 00133 Rome, Italy; 3 Department of Health Sciences (DISSAL), University of Genova, 16132 Genova, Italy; matteo.bauckneht@gmail.com (M.B.); sambuceti@unige.it (G.S.); 4 Radiation Oncology, IRCSS Azienza Ospedaliero-Universitaria di Bologna, 40138 Bologna, Italy; elisa.lodirizzini@aosp.bo.it (E.L.R.); monarifabio@libero.it (F.M.); 5 Section of Nuclear Medicine, Interdisciplinary Department of Medicine, University of Bari Aldo Moro, Piazza Giulio Cesare 11, 70124 Bari, Italy; ferrari_cristina@inwind.it (C.F.); valentina.lavelli@gmail.com (V.L.); claudia.battisti@gmail.com (C.B.); giuseppe.rubini@uniba.it (G.R.); 6 Unit of Nuclear Medicine, Department of Medicine, Surgery and Pharmacy, University of Sassari, 07100 Sassari, Italy; amarongiu2@uniss.it (A.M.); a.mura203@studenti.uniss.it (A.M.); smfnuvoli@uniss.it (S.N.); aspanu@uniss.it (A.S.); 7 Medical Oncology Unit 1, IRCCS Ospedale Policlinico San Martino, 16132 Genova, Italy; giuseppe.fornarini@hsanmartino.it; 8 Medical Oncology Unit, Ospedale San Paolo, 17100 Savona, Italy; saraelena89@hotmail.it; 9 Department of Economics & Finance, University of Rome “Tor Vergata”, 00133 Rome, Italy; alessio.farcomeni@uniroma2.it; 10 Unit of Nuclear Medicine, Biomedical Department of Internal and Specialist Medicine, University of Palermo, 90133 Palermo, Italy; alessandra.murabito@gmail.com (A.M.); renatopatrizio.costa@gmail.com (R.P.C.); 11 Nuclear Medicine, IRCSS Azienda Ospedaliero-Universitaria di Bologna, 40138 Bologna, Italy; aber.golemi@aosp.bo.it; 12 Radiopharmacy Section, Ospedale G. Mazzini, Piazza Italia, 64100 Teramo, Italy; manlio.mascia@gmail.com; 13 Unit of Nuclear Medicine, Spirito Santo Hospital, 65100 Pescara, Italy; lauratravascio.lt@gmail.com

**Keywords:** Radium-223, mCRPC, prostate cancer, multicenter study, radionuclide therapy

## Abstract

**Background/Objectives:** The prognostic value of baseline clinical parameters in predicting the survival prolonging effect of Radium-223-dichloride (^223^RaCl_2_) for metastatic castration resistant prostate cancer (mCRPC) patients has been the object of intensive research and remains an open issue. This national multicenter study aimed to corroborate the evidence of ten years of clinical experience with ^223^RaCl_2_ by collecting data from eight Italian Nuclear Medicine Units. **Methods:** Data from 581 consecutive mCRPC patients treated with ^223^RaCl_2_ were retrospectively analyzed. Several baseline variables relevant to the overall survival (OS) analysis were considered, including age, previous radical prostatectomy/radiotherapy, number of previous treatment lines, prior chemotherapy, Gleason score, presence of lymphoadenopaties, number of bone metastases, concomitant use of bisphosphonates/Denosumab, Eastern Cooperative Oncology Group Performance Status (ECOG-PS), as well as baseline values of hemoglobin (Hb), platelets, Total Alkaline Phosphatase (tALP), Lactate Dehydrogenase (LDH), and Prostate-Specific Antigen (PSA). Data were summarized using descriptive statistics, univariate analysis and multivariate analysis with the Cox model. **Results:** The median OS time was 14 months (95%CI 12–17 months). At univariate analysis age, the number of previous treatment lines, number of bone metastases, ECOG-PS, presence of lymphadenopathies at the time of enrollment, as well as baseline tALP, PSA, and Hb, were independently associated with OS. After multivariate analysis, the number of previous treatment lines (HR = 1.1670, CI = 1.0095–1.3491, *p* = 0.0368), the prior chemotherapy (HR = 0.6461, CI = 0.4372–0.9549, *p* = 0.0284), the presence of lymphadenopathies (HR = 1.5083, CI = 1.1210–2.0296, *p* = 0.0066), the number of bone metastases (HR = 0.6990, CI = 0.5416–0.9020, *p* = 0.0059), ECOG-PS (HR = 1.3551, CI = 1.1238–1.6339, *p* = 0.0015), and baseline values of tALP (HR = 1.0008, CI = 1.0003–1.0013, *p* = 0.0016) and PSA (HR = 1.0004, CI = 1.0002–1.0006, *p* = 0.0005) remained statistically significant. **Conclusions:** In the era of precision medicine and in the landscape of novel therapies for mCRPC, the prognostic stratification of patients undergoing ^223^RaCl_2_ has a fundamental role for clinical decision-making, ranging from treatment choice to optimal sequencing and potential associations. This large Italian multicenter study corroborated the prognostic value of several variables, emerging from ten years of clinical experience with ^223^RaCl_2_.

## 1. Introduction

Complications related to bone metastases, including disabling bone pain, impaired mobility, pathologic fractures, bone marrow failure, and spinal cord/nerve root compression, represent the leading cause of poor quality of life in the end-stage of metastatic castration-resistant prostate cancer (mCRPC) [1,2].

Radium-223 dichloride (^223^RaCl_2_; Xofigo^®^; Bayer HealthCare Pharmaceuticals Inc., Hanover, NJ, USA) is a calcium-mimetic α-emitter that selectively binds to areas of increased bone turnover, such as bone metastases. Emitted α particles cause non-repairable double-stranded DNA breaks on targeted cancerous cells, inducing minimal hematological toxicity due to the short range in tissues (inferior to 100 µm) [3,4,5,6].

After the phase III clinical trial, Alpharadin in Symptomatic Prostate Cancer Patients (ALSYMPCA) showed significant improvement in overall survival (OS), delayed time to first symptomatic skeletal-related events (SREs), and palliative effect on bone pain [7]. ^223^RaCl_2_ was approved by the Food and Drug Administration in 2013 as a therapeutic option for mCRPC with symptomatic bone metastases and no evidence of visceral involvement [8].

Clinical experience has shown however a lower benefit on survival, with reported median OS between 6 and 10 months, compared to 14.9 months of the ALSYMPCA trial, probably due to the presence of unfavorable prognostic factors [9,10].

Furthermore, in 2018, the European Medicine Agency (EMA) limited the use of ^223^RaCl_2_ to mCRPC patients having more than six bone lesions and showing progression after at least two systemic therapies, as well as to cases of ineligibility for any systemic treatment. The EMA restricts the use of prostate cancer medicine ^223^RaCl_2_.

The consequent administration of ^223^RaCl_2_ in the latest phases of the disease has had a further impact on survival, making patient selection particularly challenging and identifying reliable prognostic factors a crucial clinical issue for in-depth research [11].

With this purpose, several research groups have investigated and demonstrated the prognostic role of multiple baseline parameters, ranging from age, functional rating scales, and baseline values of disease markers to bone marrow state, disease load, inflammatory parameters, and previous therapies, to name a few [12,13,14,15,16,17,18,19].

However, as the landscape of mCRPC is continuously changing with the development of novel treatment options, including radioligand therapy with Lutetium-177(^177^Lu)–PSMA (PSMA-targeted RLT) [20,21,22], both the definition of the best treatment strategy fitting to the specific characteristics of each patient and the assessment of the optimal time-sequencing of all available treatment options require continuous research and need more confirmation, according to the principles of precision medicine.

After ten years of worldwide clinical experience with ^223^RaCl_2_, we conducted a large multicenter retrospective study with the aim of corroborating the prognostic relevance of multiple baseline variables by analyzing data collected from eight different Italian Nuclear Medicine Units.

## 2. Materials and Methods

### 2.1. Study Design

The study was carried out according to the ethical principles of the 1964 Declaration of Helsinki and its later amendments and was approved by the local ethical committee of all adhering centers. Written informed consent, including the use of anonymized data for research purposes was signed by each patient at the time of the first ^223^RaCl_2_ administration.

This retrospective Italian multicentric study included consecutive mCRPC patients treated in eight Nuclear Medicine Units from July 2015 to the time of the analysis in November 2024.

All patients had a diagnosis of mCRPC with symptomatic bone metastases, the absence of visceral metastatic involvement except for malignant lymphadenopathies with less than 3 cm in the short-axis diameter, adequate hematological, hepatic and renal function [23], and no inflammatory bowel disease (IBD).

They were eligible for ^223^RaCl_2_ therapy based on the criteria in force at the time of their evaluation for enrollment [12,24,25], and continued androgen deprivation therapy (ADT) during ^223^RaCl_2_. Receiving at least one cycle of ^223^RaCl_2_ therapy was mandatory for study inclusion.

According to the therapy regimen, an intravenous injection of 55 KBq/kg of body weight of ^223^RaCl_2_ is administered intravenously every 28 days for 6 scheduled cycles [26].

For pain control, conventional analgesics and glucocorticoids were administered as prescribed by the best standard of care. No data about the specific type of analgesics, as well as the use of opioids, was available. Current guidelines were followed to address any potential toxicities that occurred during treatment.

The eight centers retrospectively collected baseline variables that were relevant for OS analysis. Specifically, age, previous primary treatment (radical prostatectomy/radiotherapy), the number of previous treatment lines, prior chemotherapy, Gleason score, the presence of lymphadenopathies, the number of bone metastases, concomitant use of bisphosphonates/Denosumab, Eastern Cooperative Oncology Group (ECOG) Performance Status (PS) score, as well as baseline values of hemoglobin (Hb), platelets, Total Alkaline Phosphatase (tALP), Lactate Dehydrogenase (LDH) and Prostate-Specific Antigen (PSA), were taken into account for the statistical analysis.

OS was established from the date of the first cycle of ^223^RaCl_2_ until the date of death, last ^223^RaCl2 administration, or last follow-up contact.

### 2.2. Statistics

The data are expressed as mean plus or minus standard deviation, or median plus or minus Median of Absolute Deviations (MADs) where appropriate. Quantiles of survival were estimated through the Kaplan–Meier product limit estimator. The relationship between baseline covariates and the time-to-event endpoint was assessed using univariate and multivariable Cox regression models, adjusted for possible center-specific effects. Robust standard errors were computed accordingly. The final multivariable model was selected using a forward stepwise procedure based on the Akaike Information Criterion (AIC). A sensitivity analysis was also performed, showing robustness to the criterion used for model selection. The threshold for statistical significance was established at 5% before the analysis. All analyses are performed with R software, version 4.3.3.

## 3. Results

### 3.1. Patients’ Baseline Characteristics

The patients’ baseline characteristics are shown in Table 1.

The study included 581 consecutive mCRPC patients. Mean age was 71.99 ± 8.45 years.

Except for 83 patients, all subjects had received at least 1 previous treatment line, with more than half (63.7%) having undergone prior chemotherapy. No patients had received previous treatment with PSMA-targeted RLT.

Among 581 patients, 304 (52.3%) did not undergo any type of surgery nor radiotherapy on prostate gland, showing metastatic disease at the time of staging completion.

Approximately one-third of patients (32.2%) had known lymphadenopathies at the time of enrollment for ^223^Ra-therapy.

All subjects had symptomatic bone metastasis according to the Brief Pain Inventory (BPI) scale. A baseline bone scan was performed in all patients 2–3 weeks before the first cycle of treatment. The number of bone lesions at baseline was less than 6 in about 10% of patients, with the highest percentage of subjects having a number of bone metastasis between 6 and 20. About 30% of cases showed diffuse bone metastatic involvement, with more than 20 metastases or superscan.

More than two thirds of patients (77.6%) had an ECOG-PS of 0 or 1, with only 21.2% and 1.2% showing a value of 2 or 3, respectively. In the total cohort, most patients (63.7%) completed all six scheduled cycles of ^223^Ra-treatment, with more than half (55.4%) receiving concomitant treatment with bone protective agents.

**Figure 1:**
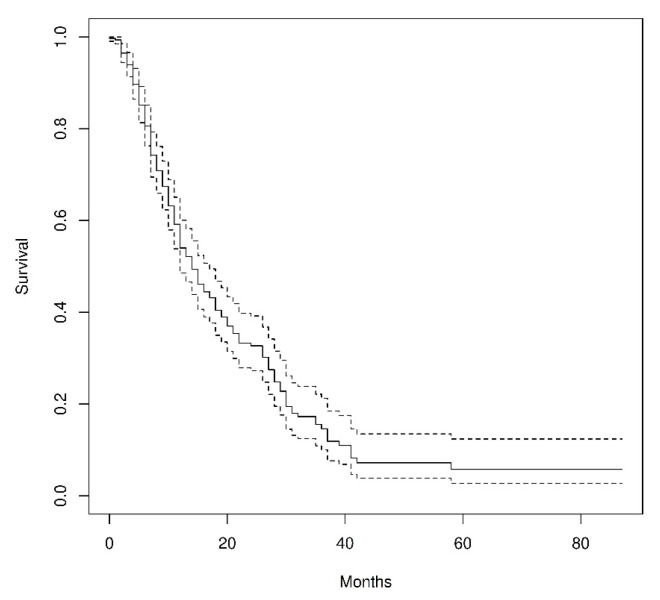
Kaplan–Meier curve for overall survival in our cohort, with 95% confidence interval in dashed lines (median survival 14 months, CI: 12–17).

Baseline blood parameters and disease markers (PSA, tALP, and LDH) are reported as mean ± standard deviation in the same table. The median OS time was 14 months (95%CI 12–17 months). Moreover, 435 out of 581 patients died at the time of the analysis. The entire survival curve is shown in Figure 1.

### 3.2. Univariate Analysis

The results of the univariate analysis are summarized in Table 2.

Considering clinical covariates in univariate models, several clinical aspects impacted OS. Higher age, number of previous treatment lines, number of bone metastases, ECOG-PS score, tALP, PSA, as well as the presence of lymphadenopathies at the time of enrollment, were independently associated with an increased risk of death.

On the contrary, higher baseline values of Hb were significantly associated with better outcomes. The results obtained for the concomitant use of bisphosphonates/Denosumab were at the brink of statistical significance, suggesting a possible effect.

All other clinical variables, specifically Gleason score, prior chemotherapy, and baseline value of platelets, did not show a significant association with OS on the univariate analysis.

### 3.3. Multivariate Analysis

When adjusting for other measures on the multivariate analysis, the number of previous treatment lines, prior chemotherapy, the presence of lymphadenopathies at the time of enrollment, the number of bone metastases, ECOG-PS, and baseline values of tALP and PSA were confirmed to be statistically significant parameters. The results of the multivariate analysis are shown in detail in Table 3.

## 4. Discussion

Prostate cancer still represents one of the most common diseases in Western countries and the second leading cause of cancer-related death for men despite an estimated 5-year survival rate of 98% for the majority of patients [27]. Once reaching the CRPC state, although an optimal condition of gonadic suppression is present (serum testosterone level of 50 ng/mL or lower), these patients will probably develop locally advanced or metastatic disease [3,28]. Furthermore, 4% of patients will have metastatic disease at initial diagnosis [29]. Radium-223 therapy has gained attention in recent years for mCRPC patients [25,30,31]. ^223^RaCl_2_ is the first radiopharmaceutical targeting bones, which was proven to improve OS in addition to bone palliation [7]. Compared to other drugs, ^223^RaCl_2_ is a favorable choice in the situation of symptomatic mCRPC and prolongs survival in both younger and older patients at the same baseline state [17]. Furthermore, ^223^RaCl_2_ was shown to be non-toxic and safe [8,10,32,33]. In addition, numerous studies have shown that patients treated with ^223^RaCl_2_ who had never received chemotherapy before suffered significantly less hematological damage than those who had [11]. In the multicenter study we are presenting, we conducted on a very large cohort of patients with a very long follow-up, and demonstrate that performing chemotherapy before therapy with ^223^RaCl_2_ is not harmful.

^223^RaCl_2_ therefore has a high therapeutic efficacy even in patients previously treated with chemotherapy. Currently, patients with mCRPC who have symptomatic bone metastases have a useful treatment option in ^223^RaCl_2_. Survival outcomes, however, are not always predictable. The pivotal phase III ALSYMPCA trial found a 30% decrease in the likelihood of mortality compared to the placebo, with median OS reported as 14.9 and 11.3 months in the experimental and control arms, respectively. This multicenter study confirms a median OS of 14 months. The issue is that there are currently no officially recognized predictive or prognostic criteria to determine which mCRPC patients might mostly benefit from ^223^RaCl_2_. However, there is a large part of research indicating that the efficacy of ^223^Ra is related to the prognostic classification of patients prior to therapy.

It was demonstrated that multidimensional approaches with several baseline variables have higher prognostic relevance and are based on optimizing patients’ selection process [34,35]. The importance of a multimodal approach was demonstrated by Halabi et al. in an article in which the correlation between various parameters and OS in patients with mCRPC undergoing first-line chemotherapy was highlighted [36]. A previously published study conducted on 430 patients proposed to integrate three prognostic biomarkers to obtain a 3-variable predictive score based on baseline ECOG PS, PSA, and serum Hb levels in mCRPC patients treated with ^223^RaCl_2_ [15]. In a subsequent retrospective multicenter study published by Bauckneht et al., it was observed that baseline levels of ECOG PS, PSA, and ALP, as well as the number of bone metastases and neutrophil-to-lymphocyte ratio (NLR), provide prognostic information in a large cohort of patients. For prognostic stratification of patients with mCRPC, the study-developed BIO-Ra score, which includes NLR, ECOG PS, number of bone metastases, ALP, and PSA, is an easy-to-use and broadly applicable tool. Higher NLR, derived NLR (dNLR), platelet-to-lymphocyte ratio (PLR), systemic inflammation index (SII), and lower lymphocyte-to-monocyte ratio (LMR) predicted worse OS. The BIO-Ra score identified three prognostic groups (36%, 27.3%, and 36.6% of patients, accordingly) with distinct median OS (31, 26.6, and 9.6 months, respectively) and is therefore able to identify subgroups of patients with distinctive survival outcomes after ^223^RaCl_2_ administration [12]. The predictive significance of baseline circulating PSA and ALP as biochemical markers of tumor extension was already shown by several studies [16,28,37,38,39,40,41,42].

Patients with a baseline ECOG PS > 2, ≥3 lines of prior systemic treatment, and lower baseline Hb values were less likely to complete all six cycles and, consequently, were less likely to benefit from ^223^RaCl_2_ therapy, according to a multicenter phase II study. Furthermore, similar to the previously described study, patients tolerated the drug ^223^RaCl_2_ regardless of prior therapy with abiraterone or enzalutamide [43]. Prior radical treatment was also shown to play a protective role in mCRPC patients treated with ^223^RaCl_2_ [16]. A poor prognosis was observed in patients with massive bone metastases (>20) on bone scan or with high tumor burden assessed by more sophisticated quantification approaches [44,45,46,47]. A multicenter study using bone disease burden quantification software demonstrated that baseline bone scan index (BSI) significantly predicts OS in mCRPC patients treated with ^223^RaCl_2_ [14].

Even in the study we presented, it is confirmed that a high disease burden leads to a worse OS. However, according to studies that analyzed the disease burden by adding bone volumes, a simple numerical count of metastases cannot be considered a reliable indicator of bone disease burden and therefore of OS. If everything is considered, these results provide support to the growing idea that using ^223^RaCl_2_ early in the mCRPC phase is a sensible and successful approach. Administering ^223^RaCl_2_ before ECOG-PS drops to 2 or worse, potentially improving the likelihood of completing the treatment plan, which is essential for receiving the most out of the treatment. In 2018, the EMA Pharmacovigilance Risk Assessment Committee, based on a review of the ERA-223 study, banned the use of ^223^RaCl_2_ in combination with abiraterone acetate plus prednisone/prednisolone in patients with mCRPC and imposed that ^223^RaCl_2_ could only be used from the third line of therapy onwards [48]. This has compromised the whole concept of using ^223^RaCl_2_ as early as possible and in the context of patients with normal blood values or low ECOG-PS [24]. Bauckneht et al. demonstrated that patients treated with ^223^RaCl_2_ after EMA restriction showed a lower mOS than those treated before restricted use (12.4 vs. 23.7 months, *p* < 0.001) [13]. The results highlighted by the present study on baseline prognostic factors remain valid: patients with positive lymph nodes for metastases before ^223^RaCl_2_ therapy have a worse OS. In many previous studies, this result has not emerged so significantly. Dizdarevic et al., in a retrospective study of 57 patients, demonstrated that the presence of coexisting lymph node disease at baseline resulted in a shorter OS compared to that found in patients without lymphadenopaties [49]. To the best of our knowledge, our multicenter study has one of the much longer follow-up periods and was performed on more than 580 patients. We can certainly confirm that, in addition to other baseline variables that have proved reliable for prognostic purposes, the presence of positive lymph nodes for disease is also a fundamental predictive factor (*p* = 0.0070).

Lastly, it is important to note that the clinical, laboratory, and imaging predictors of OS in mCRPC patients treated with ^223^RaCl_2_ therapy differ in most cases from those observed in patients undergoing PSMA-targeted RLT [50]. From the pathophysiological point of view, this divergence may reflect the distinct biological mechanisms and therapeutic targets underlying these two treatment modalities. While ^223^RaCl_2_ primarily exerts its effects through targeted alpha emission in areas of increased bone turnover, PSMA-targeted RLT focuses on delivering radioligands to PSMA-expressing tissues, which often include bone and soft tissue metastases. From the clinical perspective, understanding these distinctions may be critical for optimizing patient selection and personalizing treatment strategies. Future studies directly comparing these modalities, particularly in prospective trials, will be essential to refine our understanding and improve predictive models for both therapies.

## 5. Conclusions

In the era of precision medicine, the definition of the prognostic value of clinical variables has a crucial role in the choice of the best treatment option fitting to the characteristics of each specific patient.

This Italian multicenter study corroborated ten years of clinical experience with ^223^RaCl_2_ by analyzing data from eight different centers.

With the introduction of novel treatment options in the landscape of mCRPC, the prognostic stratification of patients might guide not only treatment choice, but also optimal sequencing and potential associations.

## Figures and Tables

**Table 1:** Patients’ baseline characteristics (*n* = 581).

Baseline Variable	Value Mean (sd)	Number of Patients (*n* = 581)	Percentage (%)
Age (years)	71.99 ± 8.45	/	/
Radical prostatectomy/RT	/		
Yes		277	47.7
No		304	52.3
N. of previous treatment lines	/		
0		83	14.3
1		179	30.8
2		152	26.2
3		109	18.7
4		58	10
Prior chemotherapy	/		
No		211	36.3
Yes		370	63.7
Gleason score	/		
Unknown		67	11.6
6		29	5
7		175	30.1
8		135	23.2
9		163	28
10		12	2.1
Presence of lymphoadenopaties	/		
Unknown		41	7
No		353	60.8
Yes		187	32.2
Number of bone metastases	**/**		
Unknown		9	1.5
≤6 (score 3)		65	11.2
6–20 (score 2)		332	57.2
≥20 (score 1)		175	30.1
ECOG-PS	/		
0		244	42
1		207	35.6
2		123	21.2
3		7	1.2
Hb (g/dL)	11.98 ± 1.54	**/**	**/**
Platelets (×10^3^/µL)	242.69 ± 89.82	**/**	**/**
tALP (U/L)	242.80 ± 296.00	**/**	**/**
LDH (U/L)	327.81 ± 237.69	**/**	**/**
PSA (ng/mL)	205.46 ± 483.56	/	/
Number of cycles of 223Ra			
1		24	4.1
2		35	6
3		43	7.4
4		50	8.6
5		59	10.2
6		370	63.7

ECOG-PS: Eastern Cooperative Oncology Group Performance Status; Hb: hemoglobin; tALP: Total Alkaline Phosphatase; LDH: Lactate Dehydrogenase; PSA: Prostate-Specific Antigen; 223Ra: Radio-223 (Radium-223).

**Table 2:** Univariate Cox regression analysis of overall survival (OS) in relation to baseline variables.

Baseline Clinical Variables	HR	CIlow	CIup	*p*
Age	1.0280	1.0100	1.0450	0.0020
Gleason score	1.0300	0.9060	1.1710	0.6520
Number of previous treatment lines	1.1700	1.0520	1.3010	0.0040
Prior chemotherapy	1.0300	0.7850	1.3510	0.8320
Presence of lymphoadenopaties	1.4680	1.1100	1.9420	0.0070
Number of bone metastases	0.6470	0.5200	0.8050	<0.0001
Concomitant use of bisphosphonates/Denosumab	1.3020	0.9980	1.6990	0.0520
ECOG-PS	1.5590	1.3250	1.8340	<0.0001
Hb	0.7640	0.6950	0.8390	<0.0001
Platelets	1.0000	0.9990	1.0020	0.6420
tALP	1.0010	1.0010	1.0020	<0.0001
PSA	1.0010	1.0000	1.0010	<0.0001

HR: hazard ratio; CI: confidence interval; ECOG-PS: Eastern Cooperative Oncology Group Performance Status; Hb: hemoglobin; tALP: Total Alkaline Phosphatase.

**Table 3:** Multivariate Cox regression analysis of overall survival (OS) in relation to baseline variables.

Baseline Clinical Variables	HR	CIlow	CIup	*p*
Number of previous treatment lines	1.1670	1.0095	1.3491	0.0368
Prior chemotherapy	0.6461	0.4372	0.9549	0.0284
Presence of lymphoadenopaties	1.5083	1.1210	2.0296	0.0066
Number of bone metastases	0.6990	0.5416	0.9020	0.0059
ECOG-PS	1.3551	1.1238	1.6339	0.0015
tALP	1.0008	1.0003	1.0013	0.0016
PSA	1.0004	1.0002	1.0006	0.0005

HR: hazard ratio; CI: confidence interval; ECOG-PS: Eastern Cooperative Oncology Group Performance Status; tALP: Total Alkaline Phosphatase; PSA: Prostate-Specific Antigen.

## Data Availability

The original contributions presented in this study are included in the article. Further inquiries can be directed to the corresponding author.
